# Identification of key autophagy-related genes and pathways in spinal cord injury

**DOI:** 10.1038/s41598-024-56683-1

**Published:** 2024-03-19

**Authors:** Zhen Shang, Weipeng Shi, Haitao Fu, Yingze Zhang, Tengbo Yu

**Affiliations:** 1 Medical Department of Qingdao University, Qingdao, 266000 China; 2 Department of Orthopedic Surgery, The Affiliated Hospital of Qingdao University, Qingdao, 266000 Shandong China; 3 Shandong Institute of Traumatic Orthopedics, Qingdao, 266000 China; 4 Department of Orthopedic Surgery, Qingdao Municipal Hospital, Qingdao, 266000 China; 5 Institute of Sports Medicine and Health, Qingdao University, Qingdao, 266000 China; 6 Department of Orthopedic Surgery, Qingdao Hospital, University of Health and Rehabilitation Sciences, Qingdao, 266000 China

**Keywords:** Spinal cord injury, Autophagy, Bioinformatics analysis, Therapeutic target, Computational biology and bioinformatics, Diseases, Risk factors

## Abstract

Spinal cord injury (SCI) can cause a range of functional impairments, and patients with SCI have limited potential for functional recovery. Previous studies have demonstrated that autophagy plays a role in the pathological process of SCI, but the specific mechanism of autophagy in this context remains unclear. Therefore, we explored the role of autophagy in SCI by identifying key autophagy-related genes and pathways. This study utilized the GSE132242 expression profile dataset, which consists of four control samples and four SCI samples; autophagy-related genes were sourced from GeneCards. R software was used to screen differentially expressed genes (DEGs) in the GSE132242 dataset, which were then intersected with autophagy-related genes to identify autophagy-related DEGs in SCI. Subsequently, the expression levels of these genes were confirmed and analyzed with gene ontology (GO) and the Kyoto Encyclopedia of Genes and Genomes (KEGG). A protein–protein interaction (PPI) analysis was conducted to identify interaction genes, and the resulting network was visualized with Cytoscape. The MCODE plug-in was used to build gene cluster modules, and the cytoHubba plug-in was applied to screen for hub genes. Finally, the GSE5296 dataset was used to verify the reliability of the hub genes. We screened 129 autophagy-related DEGs, including 126 up-regulated and 3 down-regulated genes. GO and KEGG pathway enrichment analysis showed that these 129 genes were mainly involved in the process of cell apoptosis, angiogenesis, IL-1 production, and inflammatory reactions, the TNF signaling pathway and the p53 signaling pathway. PPI identified 10 hub genes, including CCL2, TGFB1, PTGS2, FN1, HGF, MYC, IGF1, CD44, CXCR4, and SERPINEL1. The GSE5296 dataset revealed that the control group exhibited lower expression levels than the SCI group, although only CD44 and TGFB1 showed significant differences. This study identified 129 autophagy-related genes that might play a role in SCI. CD44 and TGFB1 were identified as potentially important genes in the autophagy process after SCI. These findings provide new targets for future research and offer new perspectives on the pathogenesis of SCI.

## Introduction

Spinal cord injury (SCI) is a devastating neurological injury that can cause motor sensory dysfunction and severely impact a patient's quality of life. Estimates of the global incidence of SCI range greatly, from approximately 3 in 1,000,000 to 2 in 10,000^1^. SCI is characterized by two pathological stages: primary injury and secondary injury^2^. Primary injury refers to the irreversible damage caused by a direct physical injury, resulting in neuronal and cell death in the spinal cord tissue and the disruption of the blood–brain barrier. Secondary injury, which occurs alongside primary injury, is characterized by tissue edema, inflammatory reactions, neuron apoptosis, glial scar formation, and sphingomyelin loss^3^. Patients with SCI face extreme physical and psychological trauma, and the expensive treatment and nursing costs place a large burden on patients, their families, and society. SCI is considered one of the top priorities in global health problems, but no completely effective treatment methods have been identified. Therefore, exploring the molecular mechanism of SCI pathophysiology is crucial.

Autophagy is a self-degradation and recycling process that is highly conserved and coordinated^4^. It mainly occurs in eukaryotic cells and can be categorized as macro-autophagy, micro-autophagy, or chaperone-mediated autophagy^5^. Autophagy separates misfolded proteins, damaged organelles, and other substances and fuses them into lysosomes, resulting in their degradation^6^. Autophagy is highly activated in the early stage of SCI, but its specific role in SCI is still debated. Some studies have reported that autophagy can inhibit cell death and apoptosis after SCI, promoting functional recovery, whereas others suggest that autophagy plays a role in neuronal death after SCI^7–9^.

Considering these conflicting results, further exploration of the specific mechanism of autophagy activation in SCI is crucial. Despite the growing body of research on the role of autophagy in SCI, few studies have analyzed key genes and pathways using bioinformatics methods. Examining the expression of autophagy-related genes in spinal cord tissue after SCI can help elucidate the molecular mechanism of autophagy. By analyzing the GSE132242 dataset in the Gene Expression Omnibus (GEO) database with bioinformatics technology, we identified key genes and potential pathways related to autophagy after SCI, providing new targets for the diagnosis and treatment of this condition.

## Materials and methods

### Data collection and analysis

This study searched the GEO databas. The GSE132242 dataset was used as the test dataset, and the GSE5296 dataset was used as the validation dataset. The GSE132242 dataset comprised four SCI mouse samples and four spinal cord tissue samples from a sham operation group. The GSE5296 dataset comprised three SCI model samples and three spinal cord tissue samples from a sham operation group.

### Data preprocessing and screening of differentially expressed genes (DEGs)

For the GSE5296 dataset, we used the R software package "affy" to normalize and log2 transform the downloaded raw data. For the GSE132242 dataset, the software package “getGEO” was used to acquire the expression data and platform file and we take the average of the same genes. Next, we employed the R software package "limma" to identify the DEGs between the SCI and control samples, with significance thresholds of adjusted P < 0.05 and │log2FC|> 1. Finally, we utilized the online tool "sangerbox" (http://www.sangerbox.com/login.html) to generate the heatmap cluster and volcano plots for the DEGs.

### Acquisition of autophagy-related genes

GeneCards is an extensive searchable database of genes that provides information about nearly all known genes (https://www.genecards.org/). We downloaded all genes related to autophagy from the database, calculated the median of all genes based on the "score," and then identified genes that exceeded twice the median for inclusion in our follow-up study.

### Differential expression analysis of autophagy-related genes

Autophagy-related DEGs were identified as genes that overlapped between the DEGs and autophagy-related genes. Next, the number of overlapping genes was visualized with a Venn diagram created using web tools (http://bioinformatics.psb.ugent.be/webtools/Venn/), and the "ggplot2" package was used to generate a heatmap.

### Correlation analysis of autophagy-related DEGs

The Spearman correlation coefficient in the "corrplot" R package was used to determine the correlation between autophagy-related DEGs in SCI.

### Gene Ontology (GO) and Kyoto Encyclopedia of Genes and Genome (KEGG) analyses of autophagy-related DEGs in SCI

The GO function is divided into three categories: biological processes (BP), cellular components (CC), and molecular functions (MF), while the KEGG pathways explain the primary functions of genes at the molecular level. The "cluster profiler" and "GOplot" R software packages were used to perform GO and KEGG pathway enrichment analyses and visualizations for all autophagy-related genes, and the adjusted P-value, was considered the threshold for significant enrichment. The results of the top 10 enriched categories were visualized with bubble charts and bar charts.

### Construction of the protein–protein interaction (PPI) network and identification of central genes and key modules

To elucidate the functional interactions between proteins, the Search Tool for Recurring Instances of Neighbouring Genes (STRING, https://cn.string-db.org/) and Cytoscape software (https://cytoscape.org/) were utilized. Specifically, the "Betweenness" algorithm in the "CytoNCA" plug-in was used to analyze all genes and construct a circular PPI network based on the "Betweenness" score. Subsequently, the "MCODE" plug-in was used to cluster the gene network and identify key sub-network modules. Lastly, the "CytoHubb" plug-in was employed to screen the top 10 hub genes using a variety of built-in algorithms.

### Statistical analysis

In this study, the SPSS 26.0 version was utilized for statistical analysis. First, the Shapiro–Wilk method was applied to test whether the data conformed to a normal distribution. For data conforming to a normal distribution, we employed an independent sample Student's t-test to compare the gene expression levels of samples. For non-normal distribution data, we used the Mann–Whitney U test. *P*-value less than 0.05 was considered statistically significant.

## Results

### Differentially expressed genes profiles in SCI

**Figure 1:**
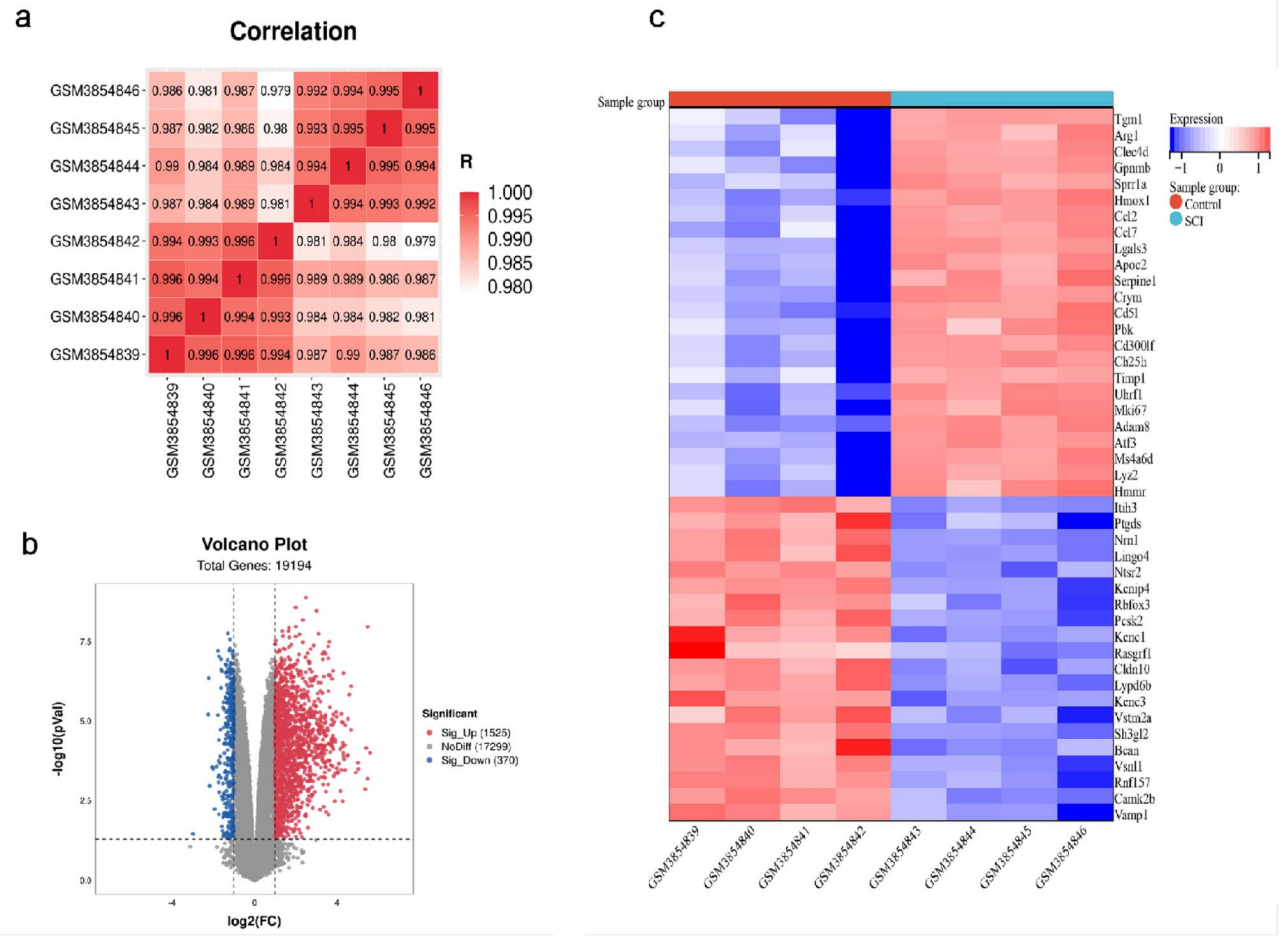
Differentially expressed genes (DEGs) in spinal cord injury (SCI) samples and control samples. (**a**) Correlation analysis of four SCI samples and four control samples. (**b**) Volcano map showing that 1525 genes were up-regulated, and 370 were down-regulated. (**c**) Heat map showing that the up-regulated and down-regulated genes.

Initially, we conducted correlation analysis on four SCI samples and four control samples, and the results indicated that the sample quality was reliable (Fig. 1a). Then, we screened 1,895 DEGs from a total of 19,194 genes. Of these, 1525 genes were up-regulated, and 370 genes were down-regulated. The findings were illustrated in the volcano map (Fig. 1b). Finally, the up-regulated and down-regulated genes were extracted and visualized with heatmaps (Fig. 1c).

### Differential expression analysis of autophagy-related genes

**Figure 2:**
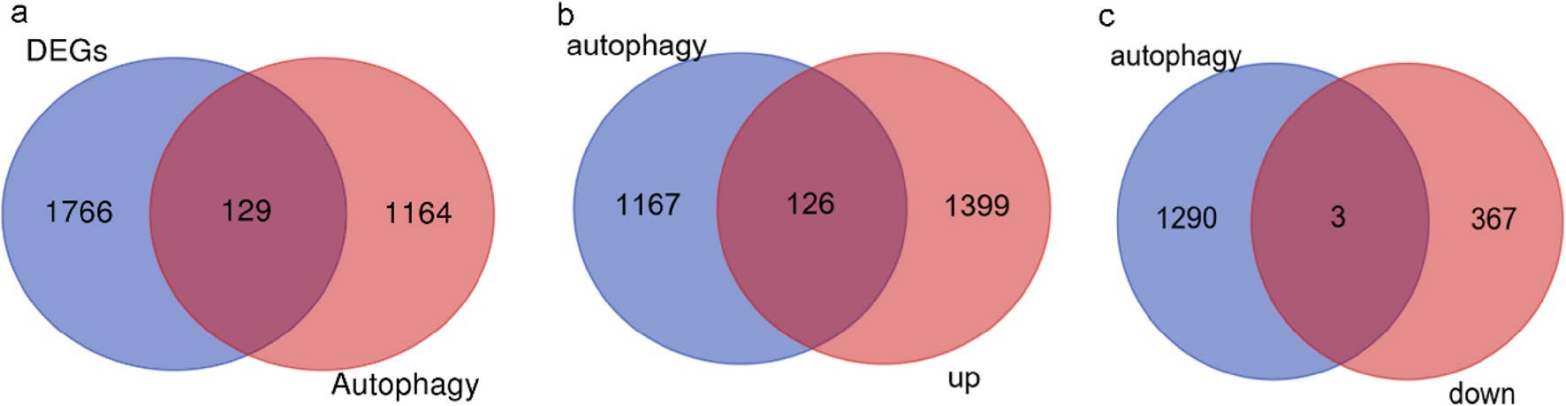
Venn diagram of autophagy-related DEGs. (**a**) Venn diagram showing 129 autophagy-related DEGs. (**b**) Venn diagram showing 126 autophagy-related up-regulated DEGs. (**c**) Venn diagram showing 3 autophagy-related down-regulated genes DEGs.

Using the two-fold median standard, we identified 1293 autophagy-related genes from the GeneCards gene database. Subsequently, we screened 129 autophagy-related DEGs, including 126 up-regulated and 3 down-regulated genes (Fig. 2a–c).

### Correlation analysis of autophagy-related DEGs

**Figure 3:**
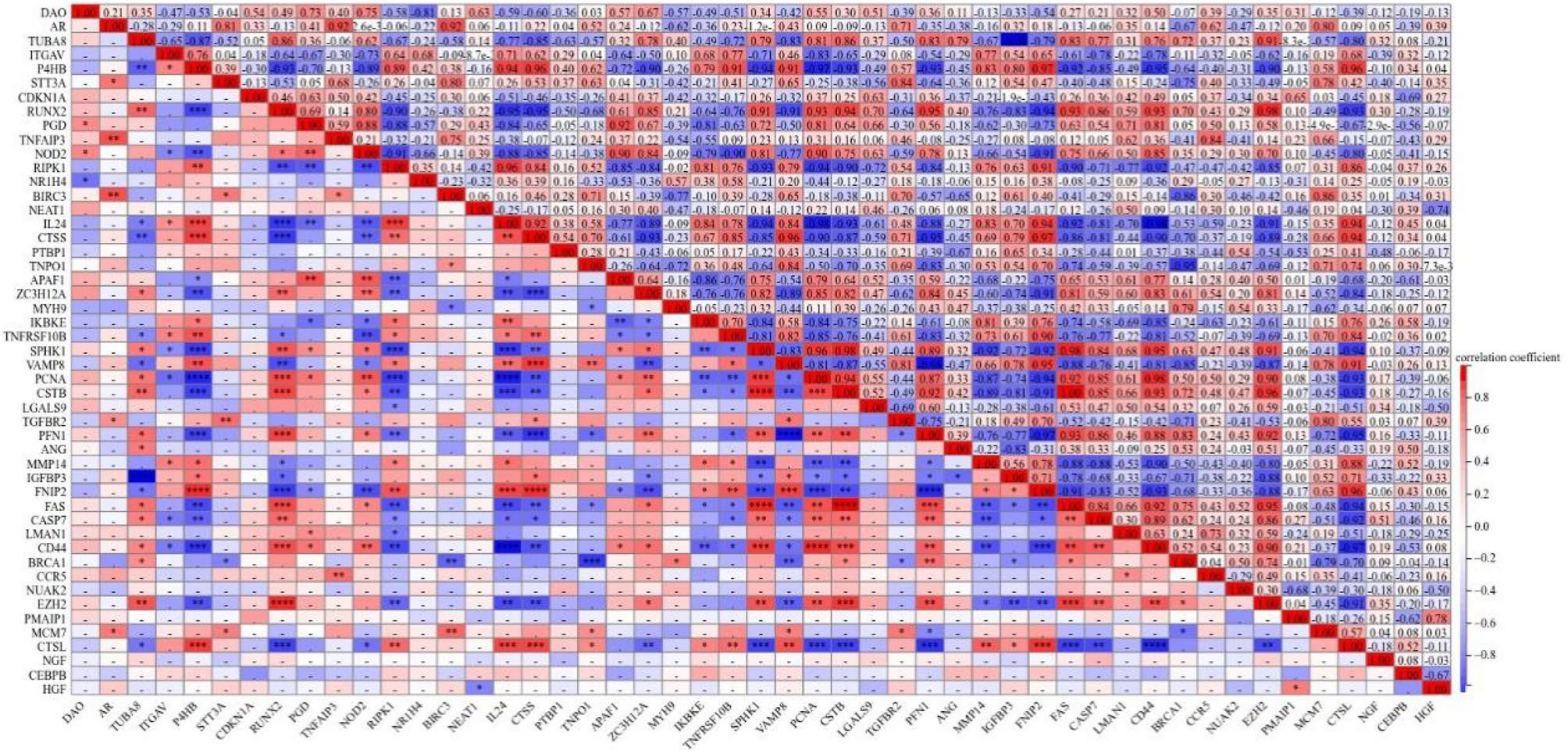
Spearman correlation analysis of autophagy-related genes that were differentially expressed in the top 50 between the SCI samples and the control samples. The results revealed various correlations among the 50 genes. FAS and CSTB showed the strongest positive correlation (Cor = 1.00). Several negatively correlated gene pairs were found among these 50 genes, including IL-24 and PCNA, VAMP8 and PNF1, and TUBA8 and IGFBP3, of these, IL-24 and CD44 had the strongest negative correlation (Cor = − 0.98).

The screening criteria for autophagy-related DEGs were P < 0.05 and │log2FC|> 1. Autophagy-related DEGs were ranked according to the value of │log2FC |, and the greater the value of │log2FC|, the greater the trend of gene difference. We performed a correlation analysis on the top 50 of the 129 autophagy-related DEGs. The results revealed different correlations among the 50 genes (Fig. 3). Notably, FAS and CSTB showed the strongest positive correlation (Cor = 1.00). Additionally, several negatively correlated gene pairs were identified among the 50 genes, including IL-24 and PCNA, VAMP8 and PNF1, and TUBA8 and IGFBP3, of these pairs, IL-24 and CD44 having the strongest negative correlation (Cor = − 0.98).

### GO and KEGG analyses of autophagy-related DEGs in SCI

**Figure 4:**
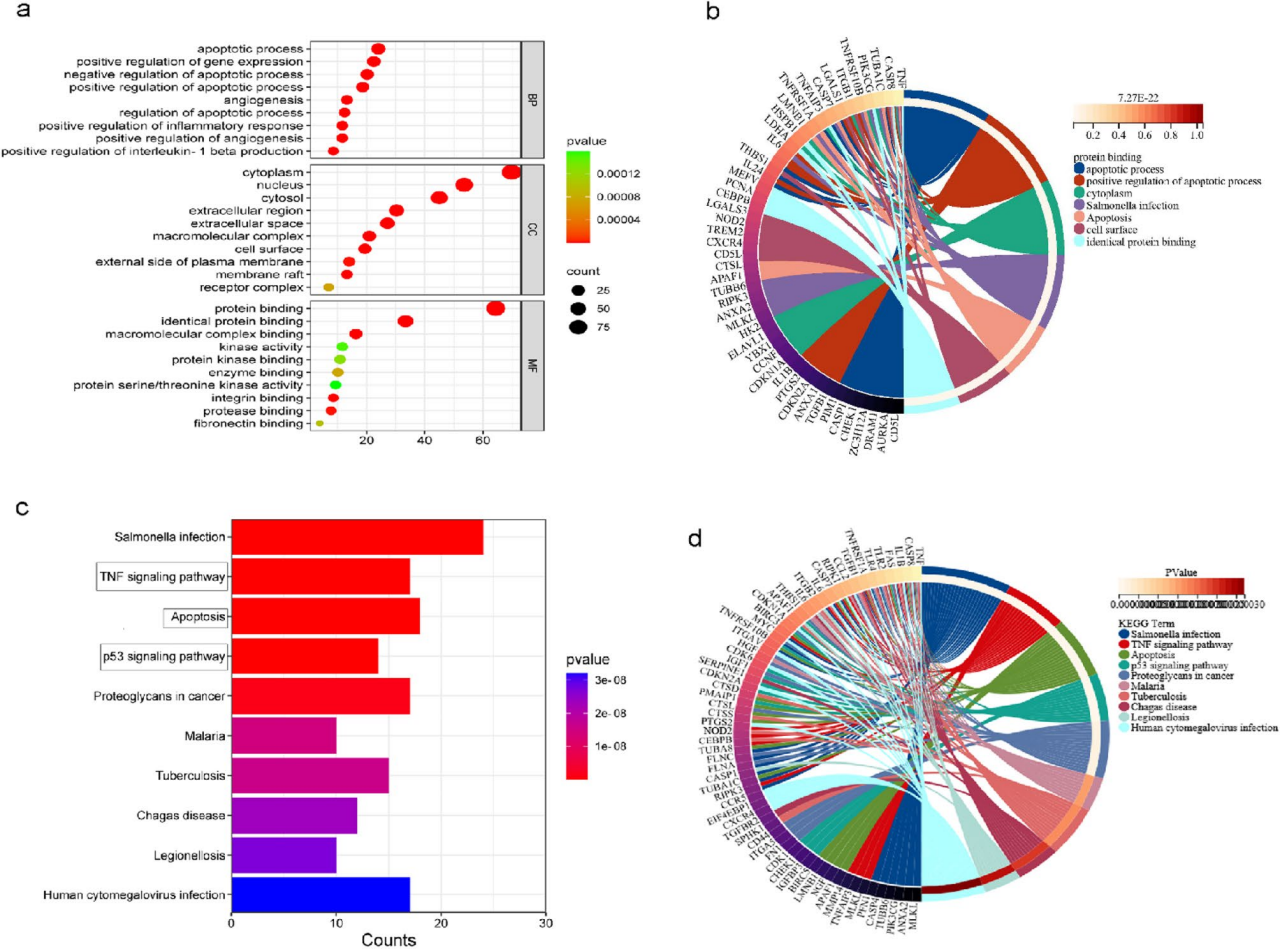
Top 10 terms in gene ontology (GO) and the Kyoto Encyclopedia of Genes and Genomes (KEGG) pathway enrichment map. (**a**,**b**) GO enrichment map visualizing the top 10 terms from biological processes (BP), cellular components (CC), and molecular functions (MF).The BP terms were mainly associated with the apical process, positive regulation of gene expression, and angiogenesis. The CC terms were mainly enriched in the cytoplasm, nucleus, and cytosol. The MF terms were mainly enriched in protein binding, authentic protein binding, and macromolecular complex binding. (**c**,**d**) KEGG pathway enrichment map showing that the 129 autophagy-related DEGs were significantly enriched in 93 KEGG pathway terms, including the tumor necrosis factor (TNF) signaling pathway, apoptosis, and the p53 signaling pathway.

To determine the biological processes and KEGG pathways of the 129 autophagy-related DEGs, we conducted GO annotation and KEGG enrichment analysis using the "clusterProfiler" R software package. We identified 674 significantly enriched GO biological process terms and selected the top 10 terms from BP, CC, and MF for visualization. The BP terms were mainly associated with the apical processes, positive regulation of gene expression, and angiogenesis. The CC terms were mainly enriched in the cytoplasm, nucleus, and cytosol. By contrast, the MF terms were mainly enriched in protein binding, authentic protein binding, and macromolecular complex binding (Fig. 4a,b, Tables 1, 2, and 3). In the KEGG enrichment analysis, the 129 autophagy-related DEGs were significantly enriched in 93 KEGG pathway terms, including the TNF signaling pathway, apoptosis, and the p53 signaling pathway (Fig. 4c,d, Table 4).

**Table 1:** Overview of the top 10 GO-BP terms.

Term	Count	%	P value	Genes
GO:0006954 ~ inflammatory response	27	20.93023	2.59E−19	CD5L, TNFAIP3, NOD2, MEFV, PTGS2, THBS1, TNF, PIK3CG, CASP7, CASP4, ZC3H12A, CCL2, OLR1, ITGAV, RIPK1, CCR5, TGFB1, ANXA1, SPHK1, NR1H4, TNFRSF1A, IL6, IL1B, TLR4, CD44, EPHA2, TLR2
GO:0006915 ~ apoptotic process	31	24.03101	8.16E−18	CD5L, IL24, TNFAIP3, TNF, AURKA, CASP7, SGPL1, CASP8, LGALS1, NUAK2, DRAM1, ZC3H12A, CHEK1, HMOX1, PMAIP1, RIPK1, APAF1, RIPK3, BIK, CDKN2A, IGFBP3, TNFRSF10B, TNFRSF1A, TGFBR2, DAP, CDK1, FAS, BIRC5, TRIB3, EPHA2, BIRC3
GO:0010628 ~ positive regulation of gene expression	29	22.48062	4.83E−17	CEBPB, SERPINE1, TREM2, BRCA1, TNF, MYC, ZC3H12A, CCL2, RIPK1, LGALS9, CCR5, TGFB1, CDKN2A, NR1H4, FN1, IGF1, NGF, RUNX2, TNFRSF1A, IL4, AR, IL6, CDK6, IL1B, CDK1, VIM, TLR4, CD44, TLR2
GO:0043065 ~ positive regulation of apoptotic process	24	18.60465	2.60E−16	TOP2A, TGFB1, ANXA1, CDKN2A, IGFBP3, IL24, TNFRSF10B, PTGS2, TNF, IL6, CASP7, CASP8, LGALS1, IL1B, CASP1, TSPO, HMOX1, PMAIP1, FAS, RIPK1, NUPR1, CTSD, TLR4, TGM2
GO:0050729 ~ positive regulation of inflammatory response	15	11.62791	2.38E−15	CEBPB, SERPINE1, MEFV, TNF, CTSS, TNFRSF1A, LGALS1, IL1B, CASP4, CASP1, RIPK1, CCR5, TLR4, TLR2, TGM2
GO:0043066 ~ negative regulation of apoptotic process	26	20.15504	4.16E−13	CDKN1A, TREM2, PTGS2, THBS1, TNF, AURKA, PLAC8, LGALS3, NUAK2, SPP1, FLNA, RIPK1, HGF, SPHK1, PLK1, NR1H4, FN1, IGF1, TNFRSF1A, IL6, CDK1, FAS, BIRC5, NUPR1, CD44, BIRC3
GO:0045766 ~ positive regulation of angiogenesis	15	11.62791	1.78E−12	GRN, HGF, SPHK1, SERPINE1, ITGB2, HSPB1, BRCA1, THBS1, HK2, TNFRSF1A, TGFBR2, LGALS3, IL1B, ZC3H12A, HMOX1
GO:0042981 ~ regulation of apoptotic process	16	12.4031	2.07E−11	APAF1, RIPK3, BIK, TNFRSF10B, NOD2, TNFRSF1A, IL6, CASP8, MYC, CASP4, CASP1, TNFAIP8L1, PMAIP1, FAS, BIRC3, TGM2
GO:0032731 ~ positive regulation of interleukin-1 beta production	11	8.527132	2.50E−11	IL6, CASP8, CASP4, CASP1, HSPB1, NOD2, MEFV, CCR5, TNF, TLR4, TLR2
GO:0001525 ~ angiogenesis	17	13.17829	4.68E−11	COL18A1, ANXA2, SERPINE1, FN1, PTGS2, PIK3CG, MMP14, CASP8, ZC3H12A, HMOX1, CCL2, FLNA, MYH9, ANG, ITGAV, ITGA5, EPHA2

**Table 2:** Overview of the top 10 GO-CC terms.

Term	Count	%	P value	Genes
GO:0005737 ~ cytoplasm	90	69.76744	1.41E−15	TOP2A, CDKN1A, CCNF, SERPINE1, HSPB1, TNFAIP3, BRCA1, TNF, HK2, PIK3CG, LMNB1, TUBA1C, PTBP1, LGALS3, CASP7, TUBB6, CASP8, LGALS1, CTSL, MYC, CASP4, ZC3H12A, CHEK1, TNFAIP8L1, CASP1, LGALS9, KPNA2, TNPO1, CCR5, IKBKE, TGM2, ANXA1, APAF1, ANXA2, RIPK3, SPHK1, PGD, RUNX2, TGFBR2, VAMP8, AR, MMP14, IL1B, BIRC5, MYH9, PLIN2, S100A4, ITGA5, NUPR1, PFN1, TLR4, BIRC3, TLR2, CSTB, CEBPB, CD5L, CXCR4, ZC3HAV1, NOD2, PTGS2, MEFV, THBS1, AURKB, AURKA, LMAN1, NUAK2, DRAM1, SPP1, EIF4EBP1, FLNA, CCL2, RIPK1, FLNC, XDH, FNIP2, LRRC25, TGFB1, MLKL, CDKN2A, PLK1, IGF1, BST2, IL6, CDK6, DAO, CDK1, FAS, VIM, TUBA8, EZH2
GO:0005576 ~ extracellular region	39	30.23256	1.04E−11	COL18A1, PON3, GRN, CD5L, SERPINE1, IL24, TREM2, IFI30, THBS1, TNF, CTSS, LGALS3, CASP7, LGALS1, CTSL, CASP4, CASP1, SPP1, CCL2, OLR1, LGALS9, XDH, CTSD, TGM2, TGFB1, ANXA1, ANXA2, HGF, IGFBP3, FN1, IGF1, NGF, IL4, IL6, IL1B, FAS, ANG, S100A4, PFN1
GO:0032991 ~ macromolecular complex	27	20.93023	4.36E−11	TOP2A, CDKN1A, CXCR4, BRCA1, NOD2, PTGS2, CASP8, MYC, CASP4, ZC3H12A, CHEK1, CASP1, EIF4EBP1, RIPK1, ANXA1, APAF1, ANXA2, RIPK3, CDKN2A, RUNX2, TNFRSF1A, AR, BIRC5, MYH9, P4HB, CD44, BIRC3
GO:0009986 ~ cell surface	25	19.37984	4.48E−11	CD5L, ITGB2, CXCR4, NOD2, THBS1, TNF, CTSS, LGALS3, LGALS1, ITGAV, CCR5, TGFB1, ANXA1, ANXA2, TNFRSF10B, TNFRSF1A, TGFBR2, BST2, VAMP8, FAS, ITGA5, TLR4, CD44, EPHA2, TLR2
GO:0045121 ~ membrane raft	17	13.17829	1.16E−10	ANXA2, ITGB2, TNFRSF10B, TNF, TNFRSF1A, TGFBR2, BST2, TUBA1C, CASP8, OLR1, FAS, RIPK1, CTSD, TLR4, CD44, BIRC3, TLR2
GO:0005829 ~ cytosol	58	44.96124	2.49E−10	CDKN1A, MCM7, IFI30, HK2, PIK3CG, LGALS3, CASP7, CASP8, LGALS1, CASP4, CASP1, TSPO, ITGAV, LGALS9, KPNA2, TGM2, ANXA1, APAF1, ANXA2, RIPK3, SPHK1, PGD, RUNX2, TNFRSF1A, TGFBR2, VAMP8, AR, MMP14, IL1B, BIRC5, MYH9, PLIN2, PFN1, CD44, BIRC3, CSTB, CXCR4, ZC3HAV1, NOD2, MEFV, AURKA, EIF4EBP1, HMOX1, PMAIP1, FLNA, RIPK1, FLNC, XDH, FNIP2, LRRC25, MLKL, TNFRSF10B, CDK6, DAO, CDK1, FAS, P4HB, VIM
GO:0005615 ~ extracellular space	35	27.13178	1.26E−08	COL18A1, CSTB, PON3, GRN, SERPINE1, IL24, THBS1, TNF, CTSS, LGALS3, CASP7, LGALS1, CTSL, CASP1, SPP1, CCL2, LGALS9, XDH, CTSD, TGFB1, ANXA1, ANXA2, HGF, IGFBP3, FN1, IGF1, NGF, TNFRSF1A, TGFBR2, IL4, IL6, MMP14, IL1B, FAS, ANG
GO:0005634 ~ nucleus	69	53.48837	7.68E−08	TOP2A, CDKN1A, MCM7, CCNF, HSPB1, TNFAIP3, BRCA1, FOXM1, LMNB1, TUBA1C, PTBP1, LGALS3, CASP7, CASP8, LGALS1, CTSL, MYC, ZC3H12A, CHEK1, CASP1, ITGAV, LGALS9, KPNA2, TNPO1, IKBKE, TGM2, ANXA1, APAF1, ANXA2, RIPK3, IGFBP3, SPHK1, RUNX2, TNFRSF1A, AR, MMP14, BIRC5, MYH9, PLIN2, TRIB3, ANG, S100A4, NUPR1, PFN1, CD44, BIRC3, CSTB, CEBPB, PCNA, CXCR4, ZC3HAV1, MEFV, AURKB, AURKA, PLAC8, NUAK2, EIF4EBP1, HMOX1, PMAIP1, FLNA, TGFB1, MLKL, CDKN2A, PLK1, NR1H4, CDK6, CDK1, VIM, EZH2
GO:0009897 ~ external side of plasma membrane	18	13.95349	1.32E−06	CD84, ITGB2, CXCR4, THBS1, TNF, TGFBR2, IL4, LGALS3, IL6, CTSL, FAS, ITGAV, ITGA5, P4HB, CCR5, TLR4, CD44, TLR2

**Table 3:** Overview of the top 10 GO-MF terms.

Term	Count	%	P value	Genes
GO:0005515 ~ protein binding	83	64.34109	1.76E−17	TOP2A, COL18A1, CDKN1A, MCM7, ITGB2, SERPINE1, TNFAIP3, TREM2, FOXM1, TNF, PIK3CG, PTBP1, LGALS3, CASP7, CASP8, LGALS1, CTSL, MYC, CASP4, ZC3H12A, CHEK1, TNFAIP8L1, CASP1, LGALS9, KPNA2, TNPO1, IKBKE, CTSD, ANXA1, ANXA2, RIPK3, HGF, NEAT1, RUNX2, TNFRSF1A, TGFBR2, RAB32, VAMP8, AR, BIRC5, MYH9, PLIN2, TRIB3, S100A4, ITGA5, NUPR1, PFN1, TLR4, CD44, EPHA2, BIRC3, TLR2, CEBPB, GRN, PCNA, CXCR4, NOD2, PTGS2, THBS1, AURKB, AURKA, PLAC8, EIF4EBP1, PMAIP1, FLNA, CCL2, RIPK1, XDH, TGFB1, MLKL, CDKN2A, BIK, PLK1, FN1, NR1H4, IGF1, IL6, CDK6, CDK1, FAS, P4HB, VIM, EZH2
GO:0042802 ~ identical protein binding	43	33.33333	3.36E−12	COL18A1, CEBPB, CD84, PCNA, HSPB1, TNFAIP3, BRCA1, ZC3HAV1, MEFV, TNF, PIK3CG, LMAN1, CASP8, LGALS1, CASP1, TNFAIP8L1, HMOX1, OLR1, RIPK1, FLNC, CCR5, XDH, IKBKE, TGM2, TGFB1, ANXA1, APAF1, MLKL, ANXA2, RIPK3, HGF, PLK1, FN1, TNFRSF10B, BST2, DAO, FAS, BIRC5, MYH9, S100A4, VIM, TLR4, TLR2
GO:0005178 ~ integrin binding	11	8.527132	8.58E−08	MMP14, IL1B, ITGB2, FN1, SPP1, MYH9, ITGAV, IGF1, ITGA5, P4HB, THBS1
GO:0002020 ~ protease binding	10	7.751938	1.53E−06	CSTB, ANXA2, SERPINE1, FN1, FAS, TNFAIP3, TNFRSF10B, ITGAV, TNF, TNFRSF1A
GO:0019899 ~ enzyme binding	13	10.07752	7.61E−05	TOP2A, PCNA, TGFB1, FN1, NOD2, BRCA1, PTGS2, AR, HMOX1, BIRC5, TRIB3, P4HB, LGALS9
GO:0001968 ~ fibronectin binding	5	3.875969	9.81E−05	CTSL, IGFBP3, ITGAV, THBS1, CTSS
GO:0019901 ~ protein kinase binding	14	10.85271	1.28E−04	CDKN1A, MLKL, CDKN2A, PLK1, ITGB2, HSPB1, NOD2, FOXM1, AURKA, MYH9, TRIB3, VIM, CCR5, CD44
GO:0016301 ~ kinase activity	15	11.62791	1.51E−04	RIPK3, SPHK1, PLK1, PIK3CG, AURKB, HK2, AURKA, TGFBR2, NUAK2, CDK6, CHEK1, CDK1, RIPK1, IKBKE, EPHA2
GO:0004674 ~ protein serine/threonine kinase activity	12	9.302326	1.59E−04	NUAK2, CDK6, RIPK3, PLK1, CHEK1, CDK1, RIPK1, IKBKE, PIK3CG, AURKB, TGFBR2, AURKA
GO:0000166 ~ nucleotide binding	26	20.15504	1.80E−04	TOP2A, MCM7, NOD2, AURKB, PIK3CG, HK2, AURKA, TUBA1C, TUBB6, NUAK2, CHEK1, RIPK1, IKBKE, TGM2, APAF1, MLKL, RIPK3, SPHK1, PLK1, TGFBR2, RAB32, CDK6, CDK1, MYH9, EPHA2, TUBA8

**Table 4:** Overview of the top 10 KEGG pathways.

Term	Count	%	P value	Genes
Salmonella infection	24	18.60465	6.94E−15	MLKL, ANXA2, RIPK3, TNFRSF10B, TNF, PIK3CG, TNFRSF1A, TUBA1C, IL6, TUBB6, CASP7, CASP8, MYC, IL1B, CASP4, CASP1, FLNA, RIPK1, FLNC, PFN1, TLR4, TUBA8, BIRC3, TLR2
TNF signaling pathway	17	13.17829	1.34E−13	CEBPB, MLKL, RIPK3, TNFAIP3, NOD2, PTGS2, TNF, TNFRSF1A, IL6, MMP14, CASP7, CASP8, IL1B, CCL2, FAS, RIPK1, BIRC3
Apoptosis	18	13.95349	1.79E−13	APAF1, TNFRSF10B, NGF, TNF, CTSS, LMNB1, TNFRSF1A, TUBA1C, CASP7, CASP8, CTSL, PMAIP1, FAS, BIRC5, RIPK1, CTSD, TUBA8, BIRC3
p53 signaling pathway	14	10.85271	1.21E−12	CDKN1A, APAF1, CDKN2A, IGFBP3, SERPINE1, TNFRSF10B, IGF1, THBS1, CASP8, CDK6, CHEK1, CDK1, PMAIP1, FAS
Proteoglycans in cancer	17	13.17829	1.31E−09	CDKN1A, TGFB1, HGF, FN1, IGF1, THBS1, TNF, CTSL, MYC, FLNA, FAS, ITGAV, ITGA5, FLNC, TLR4, CD44, TLR2
Malaria	10	7.751938	1.45E−08	IL6, TGFB1, IL1B, HGF, ITGB2, CCL2, THBS1, TNF, TLR4, TLR2
Tuberculosis	15	11.62791	1.61E−08	CEBPB, TGFB1, APAF1, SPHK1, ITGB2, NOD2, TNF, CTSS, TNFRSF1A, IL6, CASP8, IL1B, CTSD, TLR4, TLR2
Chagas disease	12	9.302326	2.34E−08	IL6, CASP8, TGFB1, IL1B, SERPINE1, FAS, CCL2, TNF, TLR4, TNFRSF1A, TGFBR2, TLR2
Legionellosis	10	7.751938	2.70E−08	IL6, CASP7, CASP8, APAF1, IL1B, ITGB2, CASP1, TNF, TLR4, TLR2
Human cytomegalovirus infection	17	13.17829	3.20E−08	CDKN1A, CDKN2A, CXCR4, PTGS2, TNF, TNFRSF1A, IL6, CASP8, CDK6, MYC, IL1B, EIF4EBP1, CCL2, FAS, RIPK1, ITGAV, CCR5

### Construction of the PPI network and identification of central genes and key modules

**Figure 5:**
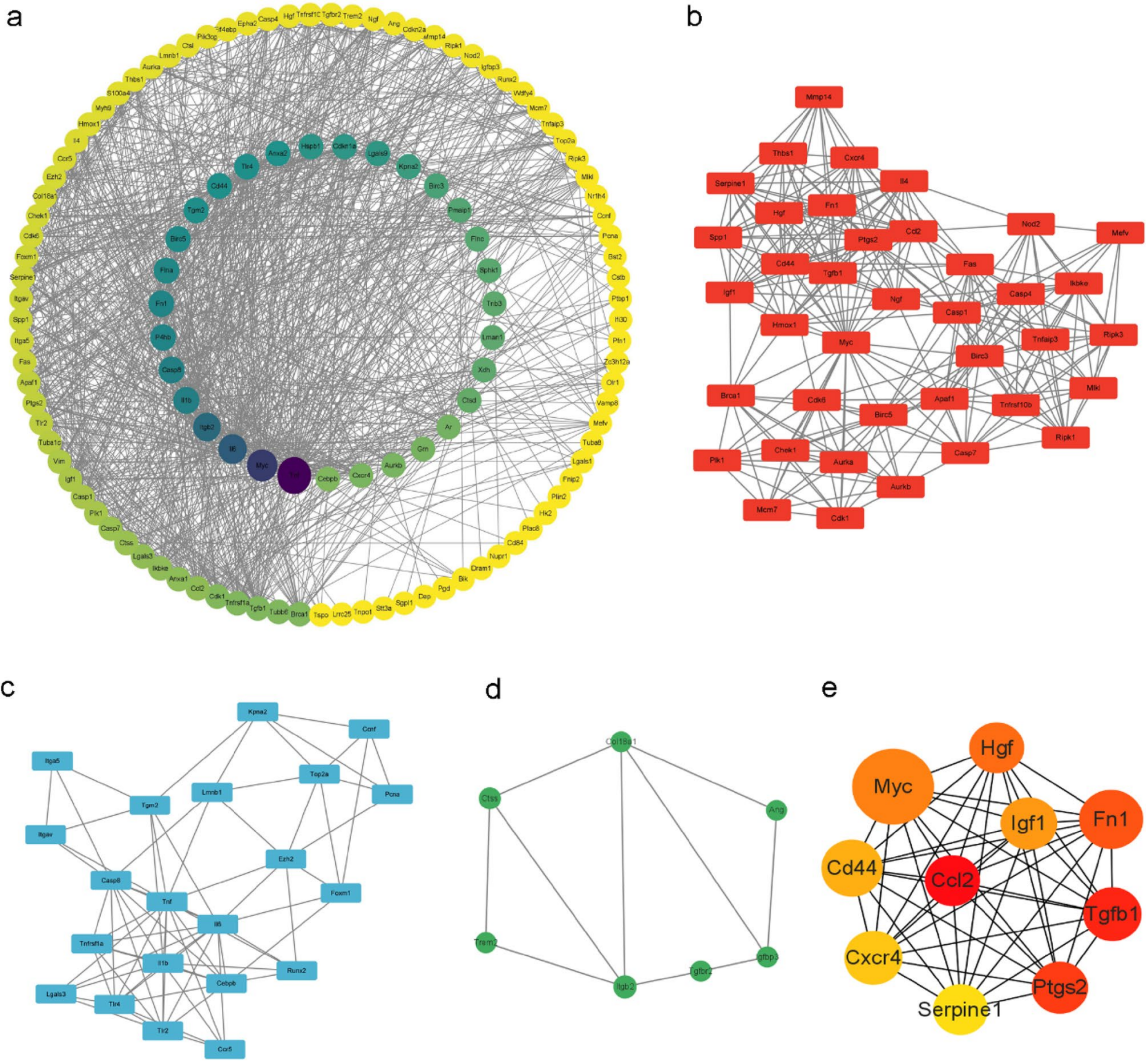
Construction of key genes networks in SCI samples and control samples. (**a**) A protein–protein interaction (PPI) network was built with the "CytoNCA" plug-in based on the Betweenness score. (**b**–**d**) MCODE plug-in showed that Cluster 1 contained 39 nodes and 271 edges, with a score of 14.263. Cluster 2 contained 21 nodes and 73 edges, with a score of 7.300. Cluster 3 contained seven nodes and 10 edges, with a score of 3.333. (**e**) CytoHubba plug-in identified 10 central genes: CCL2, TGFB1, PTGS2, FN1, HGF, MYC, IGF1, CD44, CXCR4, and SERPINE1.

To investigate the interactions between the 129 autophagy-related DEGs, we performed a PPI analysis using the STRING database and visualized the results with Cytoscape. First, we used the "CytoNCA" plug-in to build a PPI network based on the Betweenness score (Fig. 5a). In addition, we used the MCODE plug-in to identify important gene cluster modules and identified three clusters. Cluster 1 contained 39 nodes and 271 edges, with a score of 14.263. Cluster 2 contained 21 nodes and 73 edges, with a score of 7.300, while cluster 3 contained seven nodes and 10 edges, with a score of 3.333 (Fig. 5b–d). To identify central genes, we used the CytoHubba plug-in, which identified 10 central genes: CCL2, TGFB1, PTGS2, FN1, HGF, MYC, IGF1, CD44, CXCR4, and SERPINE1 (Fig. 5e).

### Cross-validation of external datasets

**Figure 6:**
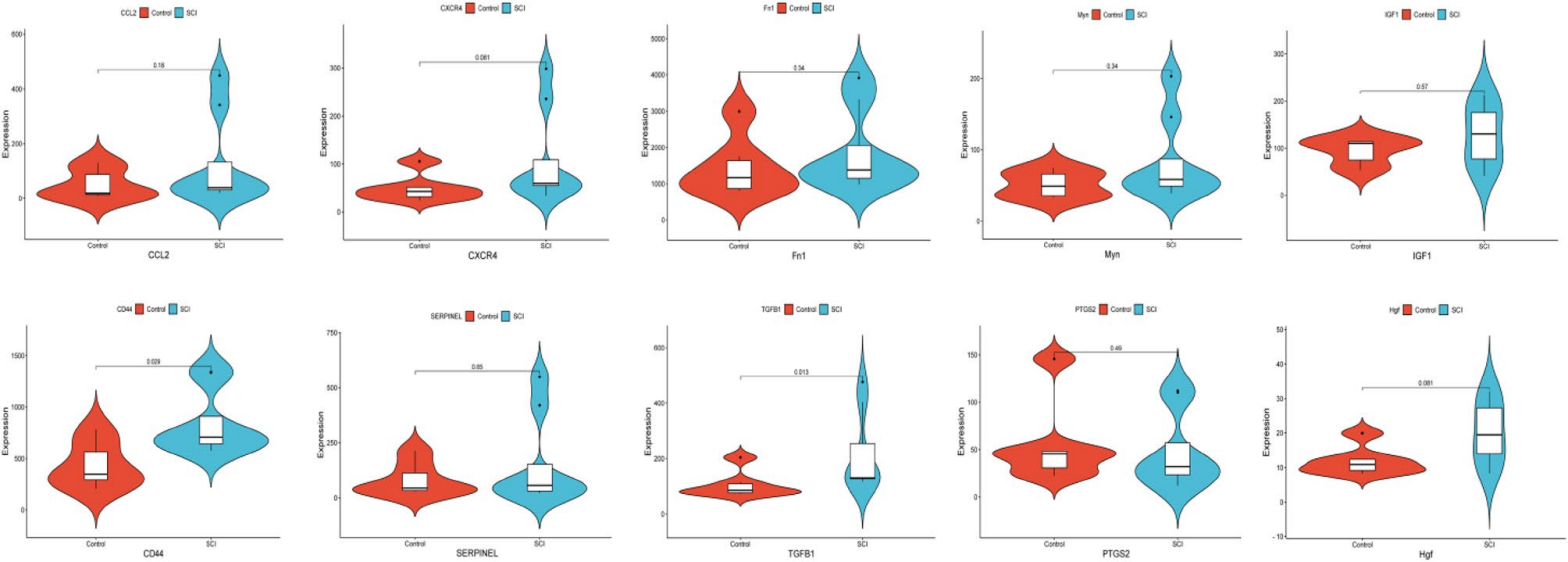
Violin Plot of 10 hub genes in SCI samples and control samples in the GSE5296 dataset. Violin Plot showing that the control group exhibited lower expression levels than the SCI group, although only CD44 and TGFB1 showed significant differences.

To verify the accuracy of our results, we conducted cross-validation with the GSE5296 dataset to examine the expression levels of the 10 key genes. We downloaded this dataset from the GEO database and selected samples with the same damage time as those in the GSE132242 dataset for analysis. Except for SERPINE1, the expression levels of the nine remaining key genes were similar to those in the GSE132242 dataset. Specifically, the control group exhibited lower expression levels than the SCI group, although only CD44 and TGFB1 showed significant differences. The expression levels of the remaining seven key genes did not show a significant difference between the two groups (Fig. 6).

## Discussion

The results of the GO analysis revealed that the 129 autophagy-related DEGs mainly functioned in the cytoplasm, nucleus, and cytosol. Their molecular functions were related to protein binding and macromolecular complex binding. These genes were involved in apoptosis, angiogenesis, and the regulation of IL-1β production and the inflammatory response.

Additionally, the KEGG enrichment analysis demonstrated that these genes were mainly enriched in the TNF signaling pathway, apoptosis, and the p53 signaling pathway. The TNF and the p53 signaling pathways are mainly associated with the neuroinflammatory reaction and the apoptosis of cells and neurons in SCI. These series of analysis results confirmed that autophagy played a crucial role in SCI by regulating inflammatory response and apoptosis and might also impact angiogenesis. p53 is a crucial regulator of apoptosis, and many apoptosis-related molecules exert their effects through p53, resulting in a complex process^10^. A prior study has identified p53 and Bax-dependent cell apoptosis induced by DNA damage as the main cause of spinal motor neuron death after nerve avulsion^11^. Another study has reported that p53-mediated spinal cord mitochondrial apoptosis induced by DNA damage is an essential mechanism of cell death after SCI^12^. Moreover, p53 is involved in cell survival and axon growth, indicating that it is a critical factor influencing functional recovery after SCI and plays a vital regulatory role in neurite outgrowth^13^. Additionally, SIRT1 may also inhibit SCI cell apoptosis by regulating the p53 signaling pathway^14^.

In recent years, as research on Traditional Chinese Medicine has expanded, scholars have discovered that Schisandrin B can reduce the inflammatory response, oxidative stress, and apoptosis of in SCI by inhibiting the p53 signaling pathway^15^. Similarly, Buyang Huanwu Decoction treats SCI by regulating the p53 signaling pathway^16^. These findings support the results of our study, indicating that the p53 signaling pathway plays an essential role in SCI.

This study found that IGF1 was enriched in the p53 signaling pathway. Previous research has demonstrated that IGF1 inhibited autophagy by activating the PI3K/Akt/mTOR signaling pathway, which promoted functional recovery after SCI in rats. However, it is unclear whether IGF1 regulates autophagy through the p53 signaling pathway and contributes to the pathogenesis of SCI; therefore, further investigation is necessary^17^.

It is well-established that TNF plays a pivotal role in the inflammatory response following SCI by inducing cytokine and chemokine expression^18^. Another study has observed that the TNF signaling pathway remained activated throughout the course of SCI, with stronger activation during the early stages^19^. On the first day after the injury, a combination of TNF, recombinant IL-6, and IL-1 at the lesion site led to the recruitment and activation of microglia and macrophages. However, by the fourth day, TNF administration reduced the activation of microglia and the size of the lesion area, suggesting that TNF plays different roles at different time points after SCI^20^.

This study found that two hub genes (CCL2 and PTGS2) were found to be enriched in the TNF signaling pathway. CCL2 is an important chemokine that regulates autophagy and responds to various physiological and pathophysiological stimuli by activating autophagy. The inhibition of CCL2 expression and the PI3K/Akt/mTOR signaling pathway can activate autophagy, effectively reducing neuronal apoptosis after SCI^21^. In another study, macrophage migration inhibitory factor inhibitors improved the motor function of rats' hind limbs by reducing the microglia and macrophages recruited by CCL2 at the injury site^22^.

Recent studies based on bioinformatics analysis have demonstrated that PTGS2 is related to iron death and immune infiltration after SCI^23,24^. Our research suggests that PTGS2 affects the functional recovery of mice after SCI by regulating autophagy, but the specific mechanism of action requires further investigation. Moreover, the GO and PPI analyses revealed that several genes might contribute significantly to the pathophysiology of SCI. For example, autophagy-related DEGs identified through GO analysis could regulate the production of IL-1β, the main mediator of inflammation, which plays a harmful role in SCI. Inhibiting IL-1β can have protective effects in SCI^25^. The up-regulation of CD44 after SCI contributes to cell adhesion and glial cell attraction, promoting SCI injury repair^26^. The SDF-1/CXCR4 interaction recruits exogenous mesenchymal stem cells into injured spinal cord tissue, which may enhance nerve regeneration. Furthermore, the CXCR4 signaling pathway is involved in the migration of Schwann cells from the peripheral nervous system to the central nervous system after SCI, improving motor function^27,28^. HGF is endogenously produced in the spinal cord of rats after SCI^29^ and gradually increases during the first week after injury, remaining at a high level in the short term. HGF helps reduce the extent of SCI and improve functional recovery by exhibiting anti-inflammatory, anti-apoptotic, angiogenic, anti-fibrotic, and neurogenic properties in transplanted neural stem cells (NSCs)^30^. TGF-β is significantly upregulated by microglia and macrophages at the epicenter, rostral, and caudal areas after SCI and plays a crucial role in regulating nerve regeneration^31^. It modulates neurite growth, promotes glial scar formation, and interacts with immune cells to mediate inflammation and the immune response induced by nerve injury^32^. The formation of the glial scar is attributed to chondroitin sulfate proteoglycan (CSPG). Up-regulated TGF-β after SCI can inhibit the autophagic flux, enhance the secretion of CSPG, and impair nerve regeneration. Targeted inhibition of TGF-β can restore the autophagic flux, reduce the formation of the glial scar, and the recovery of spinal cord function^33^.

This study has several limitations that need to be acknowledged. First, we only used the latest dataset for our analysis, resulting in a limited sample size and possible deviations in our results. Second, the validation dataset was published in July 2006, and errors due to technical reasons are unavoidable. Additionally, different SCI operation methods may lead to different results. Third, further study of the potential mechanism of the selected hub genes is limited because of a lack of in vivo and in vitro experiments.

## Conclusion

We screened 129 autophagy-related DEGs through bioinformatics analysis and identified vital pathways related to these genes. Additionally, we identified 10 hub genes including CCL2, TGFB1, PTGS2, FN1, HGF, MYC, IGF1, CD44, CXCR4, and SERPINE1. After multiple validations, the results suggested that CD44 and TGFB1 as potential research and treatment targets for autophagy after SCI. The follow-up study will experimentally verify the results of this study.

## Data Availability

The datasets used and/or analysed during the current study available from the corresponding author on reasonable request.
